# Obstructive sleep apnea comorbid with insomnia symptoms and objective short sleep duration is associated with incident hypertension

**DOI:** 10.1007/s44470-025-00027-x

**Published:** 2026-01-09

**Authors:** Nikolaos Athanasiou, Slobodanka Pejovic, Alexandros N. Vgontzas, Julio Fernandez-Mendoza, Yun Li, Maria Karataraki, Edward O. Bixler

**Affiliations:** 1 Sleep Research and Treatment Center, Department of Psychiatry and Behavioral Health, Penn State University College of Medicine, 500 University Drive, Hershey, PA 17033 USA; 2 Department of Sleep Medicine, Shantou University Mental Health Center, Shantou University Medical College, Shantou, Guangdong China; 3 Sleep Medicine Center, Shantou University Medical College, Shantou, Guangdong China; 4 Department of Psychiatry and Behavioral Sciences, University of Crete, Heraklion, Crete Greece

**Keywords:** Blood pressure, Objective sleep duration, COMISA phenotypes

## Abstract

**Purpose:**

Co-morbid insomnia and obstructive sleep apnea (COMISA) poses greater cardiovascular risks than either condition alone. We investigated whether COMISA associated with insomnia with short sleep duration (ISSD) phenotype is associated with an increased risk of incident hypertension in a large random general population sample.

**Methods:**

From the 1741 participants of the Penn State Adult Cohort, 1395 were followed-up after 7.5 years and 786 did not have hypertension at baseline. Hypertension was determined by a self-report of receiving treatment for high blood pressure. Insomnia symptoms were defined as either a complaint of chronic insomnia lasting ≥ 1 year or a complaint of difficulty falling asleep, staying asleep, nonrestorative sleep, or early morning awakening. All subjects underwent 8-h in-laboratory polysomnography. Obstructive sleep apnea was defined as an obstructive apnea/hypopnea index ≥ 5 event/h. Objective short sleep duration was defined as < 6 h sleep.

**Results:**

The mean age of the study population was 47.5 ± 12.7 years and 51.3% were women. Compared to good sleepers, the highest risk of incident hypertension was in the COMISA with ISSD phenotype (OR = 4.25, 95%CI = 1.52–11.90), followed by OSA-alone (OR = 3.31, 95%CI = 1.85–5.92) and ISSD (OR = 2.27, 95%CI = 1.29–4). The insomnia with normal sleep duration phenotype alone or with OSA (COMISA) was not significantly associated with incident hypertension.

**Conclusions:**

The additive effect of COMISA on hypertension risk is associated with the ISSD phenotype, the most severe biological phenotype of insomnia. Obtaining objective sleep duration in addition to apnea/hypopnea and/or oxygen saturation indices may lead to a more accurate diagnosis and treatment of COMISA.

## Brief summary

**Current Knowledge/Study Rationale:**

COMISA, which is combination of sleep apnea and insomnia, is frequent in clinical practice, and associated with increased risk of developing cardiovascular disorders such as hypertension. We examined whether the increased hypertension risk associated with COMISA is driven by the insomnia short sleep duration phenotype (ISSD).

**Study Impact:**

This is the first longitudinal study, based on a large random general population sample, that shows that the increased hypertension risk in patients with COMISA is associated with the ISSD phenotype. In patients suffering from COMISA, objective assessment of sleep duration in addition to the traditional measures of apneas/hypopneas and/or oxygen saturation drop could lead to more precise diagnosis and effective differential treatments, the goal of personalized medicine.

## Introduction

Hypertension prevalence in the adult US population is 47.7% and is an important risk factor for cardiovascular disease [1, 2]. Two of the most common sleep disorders, obstructive sleep apnea (OSA) and insomnia, have well-established independent associations with hypertension in cross-sectional and longitudinal studies [3–8]. The co-occurrence of these two highly prevalent sleep disorders in a single patient, referred to as co-morbid insomnia and sleep apnea (COMISA), is frequent with prevalence estimates in clinical samples ranging from 18 to 42% [9]. Furthermore, 30–50% of patients with OSA report clinically significant insomnia symptoms, while 30–40% of individuals with insomnia meet the diagnostic criteria for OSA [9, 10]. COMISA is likely to be associated with an increased risk of hypertension, suggesting that the combination of the two disorders has a potentiating adverse impact on cardiovascular health [11]. In addition, a number of recent studies suggest that COMISA is associated with increased risk of cardiac disease, stroke, diabetes and increased mortality [11–16].

During the last two decades many studies from several independent groups have shown that insomnia with objective short sleep duration (ISSD) but not insomnia with normal sleep duration (INSD) phenotype is strongly associated with significant cardiometabolic morbidity, including hypertension, diabetes, cardiovascular disease, and mortality [5–7, 17–21]. This suggests that it is the combination of a complaint of insomnia with objective short sleep duration that affects adversely physical health, and that short sleep may be a useful and valid biomarker of the severity of insomnia.

Thus far, only two studies have explored whether COMISA associated with short sleep duration (i.e., COMISA with the ISSD phenotype) has an additive effect on subclinical myocardial injury and chronic kidney disease [15, 22]. Notably, these studies have largely relied on either subjective assessment of sleep duration or actigraphy rather than polysomnography (PSG), the gold standard for the assessment of physiologic sleep [15, 22]. Recent findings from a cross-sectional clinical study suggest that the higher prevalence of hypertension in COMISA patients appears to be primarily driven by the ISSD phenotype, but not the INSD phenotype [23].

To our knowledge, no study has examined longitudinally the association between COMISA with ISSD and risk of incident hypertension. Thus, the aim of this study was to investigate whether COMISA combined with the ISSD phenotype is associated with an increased risk of incident hypertension in a large random general population sample.

## Methods

### Study design and population

**Fig. 1:**
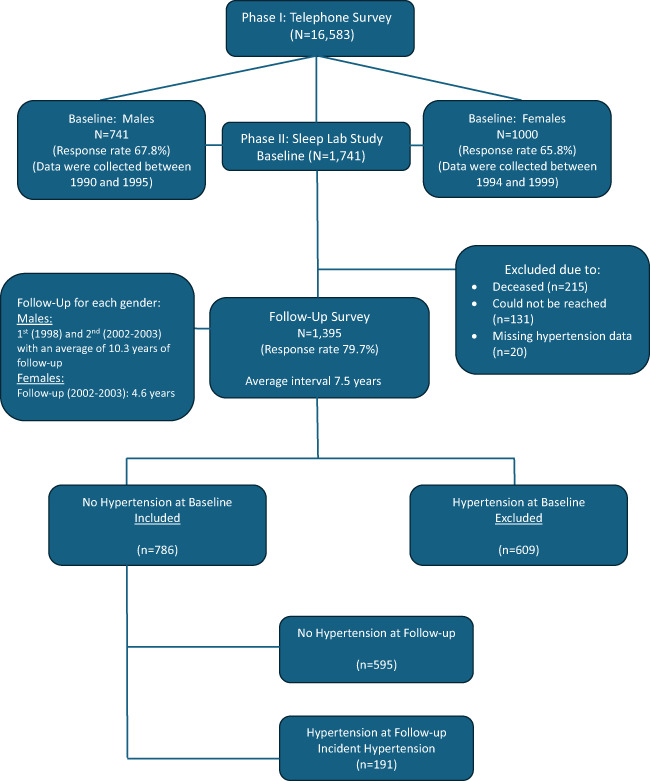
Participants’ flow in the study

The data presented were collected as part of a general population-based Penn State Adult Cohort (PSAC) prospective study investigating sleep disorders, employing a rigorous two-phase recruitment protocol to include participants across various age groups [24–26]. In the initial phase, structured telephone interviews were conducted with 4,364 men and 12,219 women (aged ≥ 20 years), residing within randomly selected households [24–26]. The questionnaire utilized in this interview collected fundamental demographic data and information related to sleep patterns. This yielded a total sample size of 16,583, with response rates of 73.5% for men and 74.1% for women. In the second phase, a stratified random subsample of 741 men and 1,000 women from the first phase underwent in-depth evaluations at our sleep laboratory, achieving response rates of 67.8% and 65.8%, respectively [24–26]. Written informed consent was obtained from all participants after providing a detailed explanation of the study’s objectives and procedures. We contrasted those subjects who were recorded in the laboratory with those who were selected but not recorded in terms of age, BMI, and prevalence of sleep disorders. There were no significant differences between these two groups on any of these variables. Of the 1,741 participants completing the laboratory assessments, 1,395 were successfully followed up after an average interval of 7.5 years through telephone interviews, with a response rate of 79.7%. Notably if we consider that 215 subjects died between baseline and follow-up, the response rate of those alive was 90.9%. In the Penn State Cohort Study, men were recruited first and women 5 years later (Fig. 1). The entire study protocol, including the follow-up, was approved by the Institutional Review Board of the university.

### Definition of incident hypertension

In this study, the presence of hypertension was defined as self-reported treatment for high blood pressure (BP), ascertained through a standardized questionnaire completed by participants during the evening of their sleep laboratory visit (baseline) or over the phone (follow-up). Among the 1,395 subjects who participated in the follow-up, 786 individuals without baseline hypertension were included in the present analysis. Of these, 191 developed hypertension during the follow-up period, while 595 remained normotensive. Data on hypertension status were unavailable for 20 participants at follow-up (Fig. 1).

### Sleep laboratory evaluation

All participants underwent a one-night 8-h evaluation in a sleep laboratory under standardized conditions in sound-attenuated, light- and temperature-controlled rooms at baseline. Bedtimes were adjusted to align with participants' usual schedules, with recordings taking place between 10:00 PM–11:00 PM and 6:00 AM–7:00 AM [24–26]. Sleep recordings were independently scored based on the Rechtschaffen and Kales criteria [27].

Percent of sleep time was calculated as the percentage of total sleep time (TST) divided by the total recorded time in bed, multiplied by 100. Based on the objectively recorded sleep time data, we reclassified the entire study sample into two ordinal groups, defined as individuals above the median percentage of sleep time ("normal sleep-duration group") and those below the median ("short sleep-duration group"). We then adjusted the cutoff point to more clinically meaningful values, creating two distinct sleep duration categories: the normal sleep-duration group, which included individuals who slept ≥ 6 h (i.e., with a sleep time percentage ≥ 75%), and the short sleep-duration group, which comprised of those who slept < 6 h during their sleep laboratory assessments [5–7, 18, 28].

Respiration was continuously monitored throughout the night using thermocouples at the nose and mouth, as well as thoracic strain gauges. Hemoglobin oxygen saturation was recorded using an oximeter attached to the finger. Apnea was considered present if a breath cessation exceeded 10 s and each apnea was categorized in terms of obstructive (chest wall movement present) or central (chest wall movement absent). In addition, hypopnea was considered present when a reduction in airflow of approximately 50% was indicated at the nose or mouth and was associated with a reduction of 4% arterial blood oxygen saturation [25, 26].

Body mass index (BMI) was calculated using measured height (in centimeters) and weight (in kilograms) recorded during the participants’ sleep laboratory visit.

### Sleep disturbances and other measurements

As part of the standardized questionnaire, the presence of sleep difficulty was systematically evaluated based on 3 levels of severity [5, 6]. First, “chronic insomnia” was defined by a complaint of insomnia with a duration of ≥ 1 year. Second, “poor sleep” was defined as a moderate-to-severe (based on a mild to severe scale) complaint of difficulty falling asleep (“Do you have difficulty falling asleep?”), difficulty staying asleep (“Do you have difficulty staying asleep?”), early final awakening (“Do you wake up in the morning earlier than desired?”), or unrefreshing sleep (“Do you still feel groggy and unrefreshed after morning awakening?”). Insomnia symptoms were defined as either a complaint of chronic insomnia or the presence of poor sleep. OSA at baseline was defined based on polysomnography findings if apnea–hypopnea index ≥ 5 events/h. COMISA was defined if both conditions were present (OSA and insomnia symptoms). Participants who did not meet the criteria for either insomnia symptoms, OSA or COMISA were categorized as normal sleepers.

Additional data collected through the standardized questionnaire included information on various physical and mental health conditions. Diabetes at baseline was defined as either being medically treated for the condition or having a fasting blood glucose level ≥ 126 mg/dL, as measured from blood drawn the morning following the sleep laboratory testing. Mental health conditions were reported based on physicians’ current or past diagnosis or treatment, including depression, suicide (thoughts, attempts), alcohol problems, drug problems and marital problems [24]. Daily consumption of alcohol (number of drinks per day) and smoking (current or past smokers), as well as demographic characteristics, including age, sex, and race/ethnicity, were also recorded.

Verbal informed consent was obtained at the follow-up, utilizing the same standardized questionnaire completed by subjects during their initial sleep laboratory visit.

### Statistical analysis

The study design incorporated oversampling of individuals at elevated risk for sleep breathing disorder and women with significantly higher body mass index to enhance the precision of risk estimates [25, 26]. To account for this sampling strategy, numerical sampling weights were created, enabling the findings to be generalized to the original target population [25, 26]. A comprehensive presentation of this sampling strategy has been presented elsewhere, including the use of the NHANES III laboratory data as the standard to adjust both the men and women in terms of sociodemographic to be representative of the national population [20, 25, 26]. We adjusted for the sampling weight in all statistical analyses, including those estimating the incidence rate of hypertension.

The demographic and clinical characteristics of the study participants were evaluated and presented as mean (standard deviation) and proportions for continuous and categorical variables, respectively. To compare these characteristics across different groups as well as with incident hypertension, analysis of chi-square test or one-way analysis of variance (ANOVA) were applied, as appropriate (Tables 1, 2 and 3). Based on prior rigorous studies [5–7, 18, 20, 23], logistic regression models were used to assess the association of sleep disturbances and objective sleep duration with incident hypertension by creating three different models as presented in Table 4. First, we examined the association of each sleep disturbance group with incident hypertension, using individuals without insomnia symptoms or OSA (normal sleepers) as the reference group (model 1). Second, we tested the interaction between each sleep disturbance group and objective sleep duration on incident hypertension (model 2). Finally, we divided the sample into seven mutually exclusive subgroups, based on the significant interactions observed in model 2: (1) good sleepers (i.e., sleep duration ≥ 6 h without insomnia symptoms or OSA, NSD), (2) short sleepers (i.e., objective short sleep duration < 6 h without insomnia symptoms or OSA, SSD), (3) OSA, (4) insomnia with objective short sleep duration (ISSD), (5) insomnia with objective normal sleep duration (INSD), (6) COMISA with the ISSD phenotype, and finally (7) COMISA with the INSD phenotype (Table 2). The OSA group was not stratified based on objective sleep duration because the interaction term was not significant for this group.

**Table 1:** Demographic, clinical, and sleep disturbances of study participants at baseline

		Incident hypertension		
Characteristics	All (*N* = 786/N* = 1102)	No (*N* = 595/N* = 906))	Yes (*N* = 191/N* = 196)	*p*
Age, years	47.5 (12.7)	46.8 (12.9)	50.8 (11.4)	< 0.001
Male, %	48.7	47.1	56.4	0.019
Race/Ethnicity (Non-Hispanic White), %	92.5	92.8	90.8	0.324
BMI, kg/m^2^	27.1 (4.8)	26.7 (4.5)	28.9 (5.7)	< 0.001
Mental health problems, %	17.8	17.9	17.3	0.859
Diabetes mellitus, %	10.0	9.4	12.8	0.155
Smoking, %	22.2	22.7	19.9	0.386
Alcohol, drinks/day	1.4 (6.5)	1.5 (7.1)	0.9 (2.6)	0.321
None, %	72.5	72.8	69.4	
1, %	10.5	9.1	16.3	
≥ 2, %	17.0	18.1	14.3	
Sleep disturbances				< 0.001
Normal sleep, %	66.7	69.9	52.8	
Insomnia symptoms, %	23.3	23.1	23.6	
OSA, %	7.5	5.3	17.4	
COMISA, %	2.5	1.7	6.2	
Objective sleep duration				0.002
≥ 6 h, %	59.3	61.4	49.5	
< 6 h, %	40.7	38.6	50.5	

*N* presents the number of participants in each group without sampling weight adjustment, and N* with sampling weight adjustment. Data are mean and (standard deviation, SD) for continuous variables and % for categorical variables, based on the weighted sampling sizes N*. BMI indicates body mass index; *COMISA* co-morbid sleep apnea with insomnia symptoms, *OSA* obstructive sleep apnea

**Table 2:** Subgroups examined in this study

OSA	INS	SSD	Name	Acronym
No	No	No	Normal sleep duration ≥ 6 h (good sleepers)	NSD
No	No	Yes	Short sleep duration < 6 h	SSD
No	Yes	No	Insomnia with normal sleep duration	INSD
No	Yes	Yes	Insomnia with short sleep duration	ISSD
Yes	No	---	Obstructive sleep apnea	OSA
Yes	Yes	No	Comorbid insomnia and sleep apnea with normal sleep duration	COMISA with INSD
Yes	Yes	Yes	Comorbid insomnia and sleep apnea with short sleep duration	COMISA with ISSD

*OSA* obstructive sleep apnea (i.e., apnea/hypopnea index ≥ 5 events per hour of sleep), *INS* insomnia (i.e., difficulty falling asleep, staying asleep, nonrestorative sleep, early morning awakening or complaint of chronic insomnia lasting > 1 year). *SSD* short sleep duration (i.e., polysomnography-measured total sleep time < 6 h)

**Table 3:** Participants characteristics across sleep disturbances/sleep duration groups at baseline

Characteristics	NSD (*N* = 238/N* = 447)	SSD (*N* = 163/N* = 288)	INSD (*N* = 152/N* = 157)	ISSD (*N* = 104/N* = 101)	OSA (*N* = 73/N* = 82)	COMISA with INSD (*N* = 19/N* = 8)	COMISA with ISSD (*N* = 37/N* = 19)	*p*
Age, years	43.3 (10.3)	53.3 (13.7)	42.5 (9.9)	51.5 (13.5)	54.4 (12.5)	46.4 (12.5)	49.4 (10.3)	< 0.001
Sex (Men), %	45.6	54.9	28.4	37.6	86.6	66.7	78.9	< 0.001
Race/Ethnicity (Non-Hispanic White, %)	93.3	91.0	94.2	94.1	90.2	75.0	94.7	0.353
BMI, kg/m^2^	25.8 (3.9)	26.7 (4.1)	28.3 (5.2)	28.2 (4.9)	29.0 (5.5)	34.4 (8.4)	32.4 (7.3)	< 0.001
Moderate/Severe OSA, %	-	-	-	-	40.2	25.0	52.6	0.400
Mental health problems, %	13.4	8.7	35.9	37.0	9.8	37.5	31.6	< 0.001
Diabetes mellitus, %	3.6	17.0	10.3	11.9	15.7	37.5	10.5	< 0.001
Smoking, %	23.7	21.5	24.5	27.7	8.4	12.5	15.8	0.045
Alcohol, drinks/day	1.9 (9.2)	1.2 (3.7)	0.6 (1.7)	0.6 (1.5)	0.9 (1.6)	0.6 (1.3)	2.2 (5.2)	0.232
None, %	71.6	69.2	84.0	78.0	61.0	75.0	52.7	
1, %	12.3	9.3	3.8	8.0	19.5	12.5	10.5	
≥ 2, %	16.1	21.5	12.2	14.0	19.5	12.5	36.8	
Incident hypertension, %	12.3	16.6	10.3	30	41.5	32.6	51.2	< 0.001

*N* presents the number of participants in each group without sampling weight adjustment, and N* with sampling weight adjustment. Data are mean and (standard deviation, SD) for continuous variables and % for categorical variables, based on the weighted sampling sizes N*. *BMI* indicates body mass index, *COMISA* co-morbid sleep apnea with insomnia symptoms, *OSA* obstructive sleep apnea, *INSD* insomnia symptoms with normal sleep duration (≥ 6 h), *ISSD* insomnia symptoms with short sleep duration (< 6 h), *NSD* normal sleep duration (≥ 6 h) without insomnia symptoms or OSA, *SSD* short sleep duration (< 6 h) without insomnia symptoms or OSA

**Table 4:** Multivariable adjusted odds ratio (95% CI) of incident hypertension associated with sleep disturbances and/or objective sleep duration

Model 1		
Sleep disturbances		
Normal sleepers	Reference	
Insomnia symptoms	1.13 (0.75–1.75)	0.520
OSA	2.98 (1.75–5.06)	< 0.001
COMISA	3.10 (1.29–7.28)	0.010
Model 2		
Sleep disturbances * objective sleep duration		
Good sleepers	Reference	
Insomnia symptoms * SD	1.99 (1.20–3.34)	0.007
OSA * SD	1.19 (0.56–2.55)	0.641
COMISA * SD	3.44 (1.27–9.33)	0.015
Model 3		
Sleep disturbances & objective sleep duration		
NSD	Reference	
SSD	1.18 (0.75–1.86)	0.467
INSD	0.66 (0.35–1.23)	0.195
ISSD	2.27 (1.29–4)	0.004
OSA	3.31 (1.85–5.92)	< 0.001
COMISA with INSD	1.80 (0.35–9.12)	0.478
COMISA with ISSD	4.25 (1.52–11.90)	0.006

All data were adjusted for sampling weight, sex, baseline age, body mass index, race/ethnicity, smoking, alcohol use, diabetes, mental health problems, follow-up duration and sex by follow-up time interaction

Model 1 examined the association of sleep disturbances with incident hypertension. Normal sleepers included individuals without insomnia symptoms or OSA

Model 2 examined the interaction effect between sleep disturbances and objective sleep duration with incident hypertension. Good Sleepers group included individuals with sleep duration ≥ 6 h without insomnia symptoms or OSA

Model 3 examined the association of the seven mutually exclusive subgroups based on the interaction between sleep disturbances and objective sleep duration with incident hypertension

COMISA, co-morbid sleep apnea with insomnia symptoms; OSA, obstructive sleep apnea; INSD, insomnia symptoms with normal sleep duration (≥ 6 h); ISSD, insomnia symptoms with short sleep duration (< 6 h); NSD, normal sleep duration (≥ 6 h) without insomnia symptoms or OSA; SSD, short sleep duration (< 6 h) without insomnia symptoms or OSA; SD, sleep duration

Logistic regression models were used for longitudinal data since we were not able to determine the exact time when the participants were diagnosed with hypertension. All the analyses were adjusted for sex, baseline age, BMI, race/ethnicity, smoking, alcohol use, diabetes, mental health problems, follow-up duration, sex by follow-up time interaction and sampling weight. A p value < 0.05 was defined as statistically significant. All analyses were conducted using IBM SPSS version 29.0 (IBM Corporation, Armonk, NY).

## Results

The overall incidence of hypertension was 18% and the average follow up period was 7.5 years.

Participants with incident hypertension were older, had a higher BMI and had lower objective sleep duration. Moreover, regarding the main characteristics of the groups, it appears that the NSD and INSD patients were younger, whereas the OSA and COMISA groups were predominantly male and had a higher BMI. The baseline demographic, sleep and clinical characteristics of the entire population as well as the corresponding characteristics of the seven sleep subgroups are presented in Table 1 and Table 3, respectively.

As shown in Table 4, Model 1 indicated that participants with OSA and COMISA were associated with a significantly higher risk (2.9 and 3.1-fold, respectively) for incident hypertension compared to normal sleepers (individuals without OSA or insomnia symptoms). In Model 2, the interaction terms between insomnia symptoms and objective sleep duration (*p* = 0.007), and between COMISA and objective sleep duration (*p* = 0.015) were statistically significant, while the interaction of OSA with objective sleep duration was not (β = 0.18, *p* = 0.641). In Model 3, participants with COMISA associated with the ISSD phenotype showed the highest risk of incident hypertension (OR = 4.25, 95% CI = 1.52–11.90, *p* = 0.006), followed by those with OSA-alone (OR = 3.31, 95% CI = 1.85–5.92, *p* < 0.001) and those with the ISSD phenotype (OR = 2.27, 95% CI = 1.29–4, *p* = 0.004), when compared to good sleepers (subjects with sleep duration ≥ 6 h, without insomnia symptoms or OSA). In contrast, neither participants with COMISA associated with the INSD phenotype (OR = 1.80, 95% CI = 0.35–9.12, *p* = 0.478), nor those with the INSD phenotype (OR = 0.66, 95% CI = 0.35–1.23, *p* = 0.195) or those with SSD (OR = 1.18, 95%CI = 0.75–1.86, *p* = 0.467) showed significantly increased risk of incident hypertension when compared to good sleepers.

## Discussion

### Key findings

This is the first prospective study to demonstrate, in a random general population sample, that COMISA combined with ISSD is associated with the highest risk of incident hypertension. These data suggest that obtaining objective sleep duration by EEG in addition to apnea/hypopnea and/or oxygen saturation indices may lead to a more accurate diagnosis and treatment of COMISA.

### Interpretation

Recent studies, both cross-sectional and longitudinal, in clinical and population-based samples, have revealed that COMISA carries a higher risk of hypertension than either of the two conditions alone [11, 12, 14, 23]. Also, two studies based on the Wisconsin Sleep Cohort and the Sleep Heart Health Study, have shown that COMISA is associated with all-cause mortality [12, 14]. Our study confirms and expands these findings in a general random population sample based on polysomnography.

### Mechanisms

Our study demonstrates that the additive effect of COMISA on incident hypertension is associated with the COMISA/ISSD phenotype. It has been shown that ISSD, but not INSD, is characterized by physiological hyperarousal, activation of the stress system (hypothalamic–pituitary–adrenal axis and sympatho-adrenal-medullary axis), impaired glucose metabolism, increased systemic inflammation and adverse health outcomes such as elevated risk of hypertension [19, 29–31]. Further, this study builds upon recent findings from a cross-sectional study in a clinical population that based on objective sleep measures, indicated that the increased risk for hypertension, observed in COMISA patients is primarily driven by the ISSD phenotype rather than by INSD [23]. Based on these data, the scientific community may consider recognizing, that in addition to OSA, COMISA associated with ISSD is a common risk factor for hypertension.

The COMISA concept has overturned the traditional view of secondary insomnia by giving equal primacy to both conditions whether symptoms or not and OSA and insomnia are regarded as two comorbid issues, in which disentangling causality in clinical practice is often difficult, if not impossible [10, 32, 33]. Indeed, even our prior studies in PSAC have shown that the presence of sleep apnea is associated with the development of insomnia symptoms, but not of chronic insomnia [34, 35]. From a theoretical standpoint OSA by inducing sleep fragmentation, frequent micro-arousals, and nocturnal awakenings, may trigger or perpetuate insomnia symptoms such as difficulties maintaining sleep or early morning awakening [10, 33]. Conversely, OSA activates the sympathetic nervous system and the HPA axis, thereby promoting a state of hyperarousal that could contribute to the development of insomnia symptoms [10, 36]. The evidence supporting a causal role of chronic insomnia in aggravating AHI and OSA severity is limited and inconsistent, whereas the findings indicating that OSA can induce insomnia symptoms are more consistent [10, 33, 34]. Finally, it appears that the combination of COMISA and ISSD may represent a distinct neurobiological phenotype i.e., HPA axis activation is primary to this phenotype whereas in OSA alone or COMISA with NSSD, HPA axis activation is secondary to the disorder.

### Clinical implications

Our study has several important clinical implications. Currently the clinical/laboratory diagnosis of sleep apnea and COMISA is primarily based on the apnea/hypopnea index and/or oxygen saturation drop and relevant symptoms. Our study suggests that beyond AHI, the assessment of objective TST in COMISA patients may serve as a valuable tool for identifying the more severe phenotype of COMISA requiring a distinct diagnostic and treatment approach. Currently, polysomnography remains the gold-standard objective method to assess TST, providing high-resolution physiological data and methodological rigor, yet its laboratory setting and resource intensity limit ecological validity by altering natural sleep patterns [37, 38]. Moreover, polysomnography is expensive and cannot be applied to millions of people suffering from insomnia and treated by primary care physicians. In contrast, actigraphy and newer wearable devices enable multi-night, longitudinal monitoring in real-world environments, thus enhancing ecological validity and scalability but at the cost of reduced specificity in detecting wake and reliance on proprietary algorithms [38–40]. This limitation emphasize the need for validation against PSG using standardized analytic pipelines [39]. Finally, there is a need for developing methods that incorporate the accuracy of measuring TST by PSG and respiratory metrics obtained by home polygraphy (HSAT).

In regard to treatment, cognitive behavioral therapy for insomnia (CBT-I) is presently recommended as the “first line” treatment. However, recent studies including a meta-analysis of seven retrospective studies suggest that CBT-I is more effective in the INSD than the ISSD phenotype (SD < 6 h) [41, 42]. In contrast, sleep inducing agents such as trazodone, which has been the second most commonly prescribed medication for insomnia in the U.S. for more than two decades, appear to be more effective in the ISSD phenotype [41, 43–45]. A recent meta-analysis by Sweetman et al. showed that CBT-I significantly reduces insomnia severity in COMISA patients, regardless of CPAP treatment status [46]. However, the authors did not address whether the effect of CBT-I is modified by insomnia phenotypes. It may be useful to tailor the approach according to the phenotype, initiating either CBT-I for the COMISA INSD phenotype or pharmacotherapy for the COMISA ISSD phenotype alongside treatment for obstructive sleep apnea. Also, when insomnia coexists with OSA, evidence indicates that insomnia symptoms are associated with reduced adherence to positive airway pressure (PAP) therapy and effective treatment of insomnia may improve adherence to PAP therapy [10]. Future large prospective randomized trials are needed to compare the differential efficacy of CBT-I versus pharmacotherapy in COMISA/ISSD patients in terms of symptom improvement and adherence to CPAP therapy.

This study has several strengths, including its longitudinal design in a randomly selected, general population-based sample spanning a broad age range (20–88 years). Also, relying on polysomnography rather than home sleep study, ensured control for sleep apnea and other primary sleep disorders. In addition, the study incorporated detailed clinical assessments, including a thorough history and physical examination and controlling for key confounders.

### Limitations

Some limitations should also be considered. First, in our study, the presence of hypertension at baseline as well as at follow-up was defined as a patient reported diagnosis or treatment of hypertension. We acknowledge that relying exclusively on "diagnosis or treatment for high blood pressure" likely underestimates true hypertension prevalence and this may bias our results. However, previous cross-sectional and longitudinal studies have applied the same definition for hypertension and have produced consistent results [6, 7, 47]. Second, the precise timing of hypertension onset could not be determined. However, our logistic regression analysis, which adjusted for follow-up time, provides evidence that the ISSD phenotype may temporarily precede the onset of incident hypertension. Third, objective sleep duration in this study was assessed based on a single night of PSG, which may not accurately reflect the participants' typical sleep duration. However, since the initial PSAC study on the association of the ISSD phenotype with hypertension, where sleep duration was determined from a single night of PSG [5], other studies based on large random general population, such as the Sleep Heart Health Study, have also established the association between the ISSD phenotype and hypertension using a single night of PSG [17, 21]. Additionally, research has shown a strong agreement in classifying insomnia with short sleep duration whether assessed over a single night or across two or three consecutive nights of PSG [7, 48]. However, the single night fixed (8 h) vs ad lib recording and certain bedtime and rise time may influence the TST cutoff for classifying objective short sleep duration. We further acknowledge that first night effect and fixed vs habitual sleep opportunity may have led some participants to have been misclassified in terms of phenotyping. Fourth, the definition of insomnia was based on self-reported symptoms diagnosis rather than a physician's diagnosis of insomnia disorder based on current clinical diagnostic criteria. In our study, we included even mild forms of OSA i.e., AHI ≥ 5 that previous studies have shown to be significantly associated with hypertension [3, 4, 47, 49]. However, we were not able to perform a sensitivity analysis with moderate or severe OSA, as our sample size was insufficient to allow for further stratification. Finally, although our analysis showed a clear dose response direction with the highest hypertension risk associated with COMISA combined with the ISSD phenotype, confidence intervals were relatively wide due to the small sample size, resulting in a potential type II error that may influence estimates, particularly in COMISA-INSD. The limitation of the small sample, especially in the COMISA INSD subgroup and the possibility of Type II error indicates that the results of our study should be replicated in studies with larger samples.

### Conclusion/Future directions

In conclusion, in our study the additive effect on incident hypertension observed in patients with COMISA is associated with the ISSD phenotype. From a diagnostic perspective, it appears that in COMISA patients, in addition to AHI and/or oxygen saturation indices, objective TST could be valuable in identifying those in the more severe patient subgroup. While PSG is the “gold standard” of measuring objective sleep duration, new methods more convenient, less expensive, and more ecologically friendly, could serve as a valid alternative for the assessment of TST. Thus, the assessment of the reliability and validity of a growing number of commercially available wearable sleep-tracking devices is an important and timely task of the scientific sleep community. Further, establishing the reliability and validity of the COMISA/ISSD phenotype would lead us to differential treatment approaches which is the goal of personalized medicine. Finally, it appears that COMISA associated with INSD phenotype may respond better to behavioral interventions whereas COMISA associated with the ISSD may respond better to pharmacology agents. We envision that in the near future, management and treatment of insomnia and COMISA can be accomplished in the office of the clinician based on a comprehensive clinical evaluation, including history and physical exam, and data from wearable sleep devices of established reliability and validity.

## Data Availability

The data that support the findings of this study are available from the corresponding author upon reasonable request.

## Abbreviations

ANOVA: Analysis of variance
BMI: Body mass index
CBT-I: Cognitive behavioral therapy for insomnia
CI: Confidence interval
COMISA: Co-morbid insomnia and sleep apnea
EEG: Electroencephalogram
EMG: Electromyogram
EOG: Electrooculogram
HPA: Hypothalamic-pituitary-adrenal axis
INSD: Insomnia with normal sleep duration insomnia
ISSD: Insomnia with objective short sleep duration
OR: Odds ratio
OSA: Obstructive sleep apnea
PSAC: Penn State adult cohort
PSG: Polysomnography
TST: Total sleep time
